# S04-1 Health benefits of different sport disciplines: an updated systematic review

**DOI:** 10.1093/eurpub/ckac093.017

**Published:** 2022-08-29

**Authors:** Pekka Oja, Sylvia Titze, Zeljko Pedisic, Danijel Jurakic

**Affiliations:** 1 UKK Institute for Health Promotion Research, Tampere, Finland; 2 University of Graz, Institute of Human Movement Science, Graz, Austria; 3 Victoria University, Institute for Health and Sport, Melbourne, Australia; 4 University of Zagreb, Faculty of Kinesiology, Zagreb, Croatia

**Keywords:** Football, Running, Golf, Chronic disease, Physical functioning

## Abstract

**Background:**

Our previous systematic review on the health benefits of different sport disciplines included 69 studies published before 2013. We found moderately strong evidence of health benefits for running and football while the evidence for other sport disciplines was either inconclusive or tenuous. The purpose of this report is to update the evidence base on published studies through 2013-2019.

**Methods:**

A systematic literature search of observational and experimental studies on the association between the participation in sport and health outcomes was conducted through electronic databases: MEDLINE via PubMed, Scopus, and Web of Science. The inclusion criteria were: participants heathy adults; type of studies cross-sectional, cohort or intervention designs; study group participation in specific sport disciplines; non-participation comparison group; health-related outcome variables in terms of mortality, morbidity, disease risk factors and function. Studies on sport injuries were excluded.

**Results:**

A total of 3909 titles and abstracts of studies published between 2013-2019 were identified for potential inclusion. The study selection process yielded 30 studies for further assessment. These studies included 26 different sport disciplines with 54 sport vs. no sport comparisons. The most prevalent sports were cycling (10 studies) and running (8 studies). Together with the previous systematic review the combined data includes 99 studies, 40 sport disciplines and 159 sport vs. no sport comparisons. Significant health benefits were found for a range of sport disciplines

**Conclusions:**

The updated systematic review provides new relevant data for an extended analysis of the health benefits of different sport disciplines and strengthens the existing evidence base for the promotion of health through participation in sports.

